# Strategies to Bridge Equitable Implementation of Telehealth

**DOI:** 10.2196/40358

**Published:** 2023-05-15

**Authors:** Allison M Gustavson, Allison A Lewinski, Ellen E Fitzsimmons-Craft, Gloria D Coronado, Sarah E Linke, Denalee M O'Malley, Alyce S Adams, Russell E Glasgow, Lisa M Klesges

**Affiliations:** 1 Center for Care Delivery and Outcomes Research Minneapolis Veterans Affairs Health Care System Minneapolis, MN United States; 2 Department of Medicine University of Minnesota Minneapolis, MN United States; 3 Center of Innovation to Accelerate Discovery and Practice Transformation Durham Veterans Affairs Health Care System Durham, NC United States; 4 School of Nursing Duke University Durham, NC United States; 5 Department of Psychiatry Washington University School of Medicine St Louis, MO United States; 6 Kaiser Permanente Center for Health Research Portland, OR United States; 7 Herbert Wertheim School of Public Health and Human Longevity Science University of California San Diego La Jolla, CA United States; 8 Department of Family Medicine and Community Health Research Division Rutgers Robert Wood Johnson Medical School New Brunswick, NJ United States; 9 Rutgers Cancer Institute of New Jersey New Brunswick, NJ United States; 10 Stanford Cancer Institute Stanford, CA United States; 11 Department of Family Medicine and Adult & Child Consortium for Health Outcomes Research and Delivery Science University of Colorado School of Medicine University of Colorado Anschutz Medical Campus Aurora, CO United States; 12 Division of Public Health Sciences Washington University School of Medicine St Louis, MO United States

**Keywords:** implementation science, equity, telehealth, equitable implementation, digital age, post pandemic

## Abstract

During the COVID-19 pandemic, the rapid scaling of telehealth limited the extent to which proactive planning for equitable implementation was possible. The deployment of telehealth will persist in the postpandemic era, given patient preferences, advances in technologies, growing acceptance of telehealth, and the potential to overcome barriers to serve populations with limited access to high-quality in-person care. However, aspects and unintended consequences of telehealth may leave some groups underserved or unserved, and corrective implementation plans that address equitable access will be needed. The purposes of this paper are to (1) describe equitable implementation in telehealth and (2) integrate an equity lens into actionable equitable implementation.

## Background

The COVID-19 pandemic catalyzed the rapid development, implementation, and scaling of telehealth, which we define for this commentary as the synchronous delivery of health care services by phone or video [1,2]. Prior to the pandemic, the implementation of telehealth was variable whereby providers, patients, and organizations could self-select to use telehealth (eg, opt-in if available). During the COVID-19 public health emergency, telehealth became the primary option for receipt of many health care services for most patients (eg, all-in). Rapid implementation often occurred with limited prior knowledge about telehealth and based on a selective sample of providers who were willing to offer it, often for only select problems that would be reimbursed, and with a selected sample of individuals who opted into using telehealth. As a result, significant gaps in equitable implementation exist given the rapid nature of the roll-out, which likely reinforces health disparities in health care access for already marginalized patient populations [3,4].

Implementation science plays a critical role in bridging the gap between the implementation of telehealth and equity. Implementation research involves understanding, evaluating, and providing strategies that enhance how much and how well telehealth is accessed, delivered, and received for the right patient at the right time [5]. This commentary addresses a gap in our understanding of *how* telehealth should be equitably implemented, adapted, and sustained to reach entire target populations (including those most in need or historically excluded) and diverse institutions (eg, high- and low-resourced institutions). It also highlights an urgent need to address unintended consequences of widespread telehealth and apply strategies to ameliorate inequity where possible. We present a broad perspective on equitable implementation of telehealth and provide discussion and recommendations through the literature, an illustrative example, and our own practical experiences. The specific purposes of this commentary are to (1) describe the importance of and nuances in equitable implementation in telehealth and (2) integrate an equity lens into actionable equitable implementation processes. Recognizing the complexities in equitable implementation of telehealth, we provide a perspective on how implementation can either exacerbate or proactively address inequity. Other authors have described inequalities in patients who have access to, use, and adhere to interventions and intervention inequalities if technology-based interventions are not equally effective for all [6]. The focus of this paper is not on the intervention but describes how adapting existing implementation frameworks have the potential to enhance equity-focused decision-making during *implementation* to facilitate equitable telehealth outcomes.

## Importance of Equitable Implementation

Implementation science extends its long focus on health equity [7-12] to play an essential role in understanding, adapting, and reevaluating the integration of telehealth [5,13]. While evidence-based interventions existed for telehealth practices in selected settings, contexts, and populations before the pandemic [14-17], the urgency with which these practices were adopted during the pandemic limited more deliberate evaluation of their expanded implementation. Of particular concern was the inability to consider the often sparse evidence available and to evaluate initial conditions of inequity and other contextual factors. Many systems adopted telehealth based on resources available, lessons learned from collaboration with other systems, and practical experience. This helped with the expediency required but did not allow for the careful consideration needed to avoid unintended consequences, including the potential to create or exacerbate inequities.

To guide *equitable implementation*, it is ideal to begin with a framework that accounts for social disadvantage and injustice [8,10,12,18,19]. Implementation frameworks [11]—particularly the Health Equity Implementation Framework by Woodward et al [7] and the Consolidated Framework for Implementation Research by Damschroder et al [20]—articulate conceptual models to understand *determinants* of health equity to better adapt interventions and implementation strategies. Other frameworks have focused specifically on digital equity [21,22]; of note is the Digital Health Equity Framework (DHEF) [23,24]. The DHEF considers the multilevel, ecological impact when digital determinants of health (how digital health technologies influence equity in health) interact with intermediate health factors (eg, environment, current health status, and health-related beliefs and behaviors) [25]. While these digital and equity-focused frameworks provide a critical foundation for identifying and measuring different factors related to health equity, we are still left with *how* to integrate this knowledge into equitable implementation strategies and evaluation of telehealth.

## Framework Consideration to Maximize Equitable Implementation of Telehealth

Our goal was to provide a broad perspective and guidance to health care systems and researchers on strategies to equitably use and evaluate the implementation of telehealth. We chose to illustrate our perspective by selecting and integrating exemplar frameworks that capture the rapid speed at which telehealth is being adopted and implemented across disciplines and health care settings, as well as contextual factors that might influence equitable outcomes. We also sought an integrated framework that could expand beyond understanding implementation determinants to describe *processes* by which equity or inequities are driven by the interaction with and context of the external and internal environments.

**Figure 1:**
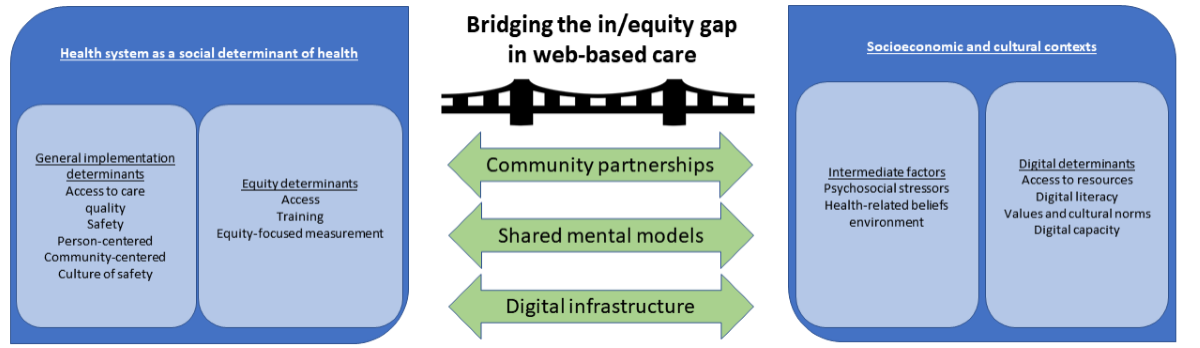
Critical bridging solutions to equitable telehealth implementation that integrate the EPIS (Exploration, Preparation, Implementation, and Sustainment) and the Digital Health Equity Framework.

With these considerations, we integrated the EPIS (Exploration, Preparation, Implementation, and Sustainment) and DHEF to encapsulate equity within the process of implementation, where EPIS guides us in moving from concept to impact and the DHEF tells us where to focus if we want to impact equity (Figure 1) [25-28]. In addition, we wanted to focus on the iterative nature of implementation and the multiphase EPIS conceptualization fit this need especially well. The rapid cycle guidance provided by EPIS is advantageous in the case of telehealth as—for the most part—exploration and preparation phases were accelerated to rapidly move to emergency implementation during the initial stages of the pandemic and limited the ability to conduct a thorough community needs assessment.

EPIS encompasses a 4-phase implementation cycle (Exploration, Preparation, Implementation, and Sustainment) and describes implementation processes, inner and outer contextual factors, and bridging factors that facilitate the interplay between inner and outer factors through each of these phases [26]. For example, internal factors may be parsed out into organizational (eg, leadership decision-making, capacity, and resources to deliver telehealth) and individual levels (acceptability, technology skills and proficiency, and literacy). External factors may include federal or state policies and reimbursement surrounding telehealth, investment in digital infrastructure (eg, technical support and equipment), and reimbursement policies around what providers can deliver telehealth and in what instances.

The DHEF adds an equity focus to the internal and external factors in EPIS [29]. The DHEF was developed to consider the multilevel health equity factors that can reduce or exacerbate disparities in access to and receipt of digital health technologies (eg, telehealth, mobile health apps, web-based health services, and wearable technologies) [23]. Importantly, the DHEF expands upon the concept that the health system is a social determinant of health, and therefore, organizations or systems need to look beyond patient-level factors to truly lead the implementation of telehealth with equity. When integrated with EPIS, we start to regroup internal and external factors into the health system as a social determinant of health and socioeconomic and cultural contexts, respectively (Figure 1). In this way, we capture both the (1) general implementation determinants (access to care, quality, and safety) [29] and equity determinants (eg, access, training, and equity-focused measurement) within the health care system and (2) the intermediate factors (eg, psychosocial stressors, health-related beliefs) and digital determinants (eg, access to digital resources, digital health literacy, and digital capacity building) in the broader societal context.

The integration of EPIS and the DHEF informs equitable implementation in telehealth by considering who can or cannot (1) access telehealth, (2) receive telehealth, and (3) deliver telehealth and *why*. An understanding of who in the population is not reached and why they may have been excluded can lead to diverse community and health system engagement and offer contextual adaptations to the telehealth clinical practice and implementation strategy.

## Bridging Solutions to Equity Across Multilevel Contexts

### Overview

EPIS contains “bridging factors,” which consider and account for the interconnectedness and bidirectional nature of movement within and across internal and external factors that shape telehealth access, receipt, and delivery [26,28,30]. Instead of exploring internal and external contexts separately, bridging factors examine the interdependence of how external forces shape health systems and vice versa. While not explicitly cited as a bridging factor in the original EPIS model, we surmise that equity is a product of critical bridging factors that tie together various levels of context (potential drivers of inequities) important to telehealth implementation. For instance, complex dynamics of oppression and injustice may be operating on multiple levels (eg, ideological, internalized, interpersonal, and institutional) that require proactive, aligned bridging strategies to overcome [31-33].

In Figure 1, we propose 3 bridging solutions whose presence supports equity of telehealth access, receipt, and delivery: community partnerships, shared mental models, and digital infrastructure. These bridging solutions connect equity concepts of the health system as a social determinant of health and socioeconomic and cultural contexts (DHEF) over the course of an implementation process (EPIS). First, fostering *community partnerships* are the backbone of successful implementation research and can create the bidirectional flow of information necessary to align telehealth goals across care systems, individuals, and national or state policies. Second, *communicating mental models* nurtures the sharing of an interrelated set of beliefs that shapes a person’s expectations for the future and how they understand the ways the world works [34,35]. When mental models are shared across systems, challenges that were seen as intractable can be resolved to achieve a common care delivery improvement goal (eg, improve access to underserved populations) [28,35]. Finally, bolstering *digital infrastructure* bridges an organization’s human and technical resources to provide telehealth with a focus on advocacy at federal or state levels to incentivize payers to reimburse telehealth and invest in digital infrastructure. In future, if rapid deployment suggests bridging factors as key considerations for the equitable implementation of telehealth. Table 1 outlines a guide to questions that promote bridging solutions for the equitable implementation of telehealth.

**Table 1:** Guide to questions that promote bridging solutions for equitable implementation of telehealth.

Bridging issues for future exploration	Question for future research
**Population**	
Patients who do not use, or minimally use, telehealth in health care delivery Patients who experience challenges to health and wellness due to sociodemographic factors Patients who experience intersecting health and sociodemographic factors	What is the impact of (*age, sex, race/ethnicity*) on telehealth reach? What are the factors of patients who do not (*receive, sustain, engage in*) telehealth? What (*resources such as interpreters, accessible devices*) are necessary for equitable implementation of telehealth? What structural factors (eg, *racism*) impact the equitable use of telehealth in a population?
**Setting**	
Community-based health settings Outpatient settings (eg, community-based clinics, large health care systems, small health care settings, rural clinics) Primary care or specialty settings	What contextual factors influence equitable delivery of telehealth at the (*person, provider, health care system*) level? What factors promote or hinder equitable implementation of (*telehealth modality*) at the (*setting*) level? In what ways does (*setting*) impact the delivery and receipt of (*telehealth modality*)? How does the implementation of telehealth differ by local or national (*policy, available infrastructure*)? How does adaptation of (*telehealth modality*) interventions differ by setting?
**Outcomes**	
Patient-centered outcomes Acceptability of telehealth (eg, access, use, sustainment) Patient and clinician satisfaction Harms and unintended consequences (eg, missed diagnoses, nonengagement of certain groups or individuals)	How are outcomes culturally or contextually defined in telehealth use? How do patient-reported outcomes differ by (*patient characteristic, subgroup*)? What patient-reported outcomes differ by (*patient characteristic, subgroup*)?

### Case Scenario

We offer a case scenario that illustrates the implementation phase of EPIS and considerations for equitable telehealth as outlined by DHEF. We first illustrate the health system as a social determinant of health and socioeconomic and cultural contexts defined by DHEF that drive the multilevel context in which the delivery of web-based physical therapy evolved over the pandemic. During the initial weeks of the COVID-19 pandemic in the United States, in-person services provided by outpatient physical therapists were immediately discontinued. Outpatient physical therapists across a multitude of health care systems and clinic networks transitioned from almost 100% in-person visits to almost 100% web-based visits in a matter of weeks [36,37]. This shift was significant in the physical therapy profession as prepandemic restrictions in the adoption of telehealth included both internal and external factors: reimbursement challenges, lack of organizational infrastructure to support web-based platforms of care, and limited provider education and training in web-based delivery of physical therapy services hampered widespread adoption of telehealth [38-40]. Additionally, before the pandemic, the physical therapy profession was already grappling with disparities in access to in-person physical therapy due to reduced staffing capacity. Staffing issues, in turn, precipitated long waitlists and high out-of-pocket costs for patients due to restrictive insurance policies for reimbursement of specialty physical therapy care [41-44]. Therefore, the quick deployment of telehealth may have further underscored unequal access for patients already experiencing challenges in receiving physical therapy care before the pandemic.

As the pandemic has progressed, so has the phased reopening of outpatient physical therapy services for in-person care. However, the value of telehealth—for example, the potential for expanded access and decreased transportation burden—has created momentum to continue the provision of physical therapy care via web-based modalities [38,45]. An operational response in some health care systems was to create threshold goals for the percentage of patients receiving in-person physical therapy care. For example, outpatient physical therapy clinics were expected to perform, say on average, 60% of visits as in-person care by a specified date in the phased reopening. Some health care systems and clinics have used additional policies that dictate a web-based visit must be initiated before an in-person visit, thus requiring the use of telehealth for entry into a physical therapy care pathway. In addition, it is important to note that the demand for these services may have increased during this period of time as well as the number of individuals seeking care due to rehabilitation after COVID-19 infection [46,47].

The context described above impacts groups at multiple levels and creates opportunities to enhance equity in the delivery of web-based physical therapy. As such, to consider further adaptations to and appropriate sustainment of web-based physical therapy care, we describe the bridging factors necessary to promote equitable implementation. First, *establishing community partnerships* is needed to engage patients, families, providers, and communities to better identify (needs assessment) who receives physical therapy (or not) when telehealth is offered and *why*. Methods to build community partnerships in implementation research offer opportunities for reflexivity and iteration, which informs strategies to ensure whether telehealth is delivered in a manner that is fair and just. For example, Miller et al [37] showed that patients reached by telehealth delivery of physical therapy during the pandemic were largely younger than 65 years, non-Hispanic White, English-speaking, commercially insured, and with few to no comorbidities. This contrasted with the distribution of patient characteristics seen for in-person physical therapy the year prior to the pandemic, many of whom were older than 65 years, Asian, non–English-speaking, noncommercially insured, and had at least 1 comorbidity. Community partnerships may enhance equitable implementation through the adaptation of physical therapy telehealth to the sociocultural context, thereby increasing the relevance of telehealth to marginalized patient populations and enhancing individual functional outcomes. Community partnerships between patients, providers, and operations are also needed to evaluate organizational capacity to provide both in-person and telehealth options based on patient needs and preferences.

Second, *communicating shared mental models* within and across systems and sectors allows groups of people delivering, receiving, or being impacted by physical therapy telehealth to be on the same page regarding equitable implementation. Telehealth delivery of physical therapy will likely persist post pandemic and adjusting how patients, providers, and systems perceive this new reality is essential to promoting equitable implementation. Discordant mental models may unintentionally hinder access to *any* modality of care such as in the case example where system or clinic policies drive the (1) proportion of telehealth versus in-person appointments available and (2) type of appointment necessary for entry into the care pathway. For example, individuals without stable internet access in a secure, private setting may be unable to engage in physical therapy if telehealth is initially required for entry into that service. Additionally, some patients may feel more comfortable receiving in-person care in a physical therapy clinic during which they can discuss sensitive topics influencing their recovery and feel less vulnerable undressing or exposing certain areas of their body for examination. Alternatively, some patients may feel more comfortable discussing sensitive topics in their own homes, thus creating an inviting atmosphere for greater sharing and conversation between patient and provider.

Convening a diverse group of community members can help build a shared mental model by asking questions such as the following: what proportion of telehealth visits per provider reaches the most patients? What is considered a successful telehealth episode of care? What criteria indicate other modalities of care be considered? Mapping clinic workflow is also essential for identifying gaps where patients may be unable or do not receive the necessary physical therapy services in a timely manner. Practice facilitation may be one strategy to allow physical therapists in a health care system to internalize approaches to ensure the right patient has access to the right modality of care at the right time [48,49]. Practice facilitation is an intervention where an external or internal facilitator interacts with multilevel stakeholders and can offer tools, resources, expertise, and guidance on strategies that address gaps and optimize workflow. Importantly, practice facilitation in the context of web-based physical therapy care can develop an internal capacity for change that can transcend the delivery of telehealth to be adaptive and receptive to evaluating and promoting equitable implementation.

Third, building *digital infrastructure* at multiple levels is necessary to ensure any patient who would benefit from physical therapy services has the option to participate in telehealth, if clinically appropriate and it aligns with patient preferences. To understand the baseline level of infrastructure, research is needed to measure (1) individual-level factors such as technology skills and proficiency, equipment availability, acceptability, and preferences for care and (2) community-level metrics such as neighborhood availability of Wi-Fi or broadband and transportation to in-person appointments. This information and ongoing evaluation inform policies and oversight of policy implementation. Building a digital infrastructure is interconnected with establishing community partnerships and communicating shared mental models as the infrastructure involves cross-sector collaborations for resources, governance, and continual monitoring. An opportunity exists to co-design or adapt aspects of digital infrastructure to better meet the needs of *all* patients who would benefit from physical therapy care, clinicians providing physical therapy, and health systems offering physical therapy services. To be truly successful, a digital infrastructure must develop a plan for transparency and sharing of data, engage the community throughout the infrastructure planning and implementation, and manage data privacy and security [50]. A strong digital infrastructure can provide the foundation to expand the equitable implementation of telehealth to other health and community services.

## Future Directions and Recommendations Post Pandemic

Deployment of telehealth will likely persist in the post pandemic era, given patient preferences for such care, emerging advances in technologies, paradigm shifts in health care professional training, and the potential to serve populations with limited potential for high-quality in-person care (eg, residents of rural areas and patients who are homebound) [21,51]. Reevaluating and adapting telehealth to promote equitable implementation is one way to identify patient groups who may be harmed by the web-based delivery of services or those who may be negatively impacted by a full return to non-telehealth delivery. We recommend evaluating the ongoing and future implementation of telehealth by (1) evaluating hybrid care models, (2) identifying multilevel barriers and facilitators to adapting technology resources that enhance access and use across diverse populations, and (3) exploring the intersectionality of telehealth access and usage with respect to age, race, ethnicity, sexual orientation, disability (including visual and hearing impairments), socioeconomic status, social determinants of health, digital health literacy and numeracy, or residence in rural or urban settings. Bridging issues outlined in Table 1 can be alleviated through bridging solutions (Figure 1): establishing community partnerships, communicating shared mental models, and building digital infrastructure.

First, establishing *community partnerships* (including those often excluded or marginalized) is the intentional and meaningful involvement of impacted community members to understand key issues and problem solving [12,52]. Feedback loops among implementation actors at multiple levels—patients, providers, clinical/health care system leadership, and policy makers—are needed to capture the barriers, facilitators, and unintended consequences to delivering or receiving telehealth, thus enabling a stronger understanding of who is impacted by telehealth delivery and how. At the organizational and policy level, assessing organizational readiness to support multiple modalities and options for care delivery is necessary to honor individual preferences for care while minimizing disruption to clinic workflow [53,54]. We acknowledge that considerable time, support, and relationship building with impacted community members is needed when conducting equitable implementation research. The time, effort, burden, and compatibility with workflow need to be periodically evaluated and modified as needed to make equitable implementation of telehealth sustainable. Future areas of evaluation highlight the need to describe how patient and multilevel partners and contextual factors can impact the uptake and adoption of telehealth through mediation or moderation. Specifically, future work is necessary to examine the uptake and adoption of telehealth by population and setting (high vs low resourced) to promote equitable use of telehealth in health care. Second, *shared mental models* between those impacted by telehealth adoption can determine the level of telehealth they are willing to accept and what changes—such as adaptations to implementation—they may consider for enhanced equity [34,35]. Adaptations are also an important aspect to maximizing equitable implementation by minimizing unintended consequences. For example, systems or clinics may need to integrate assessments of health/technology literacy/numeracy [55-57] into routine clinical care and then create or adopt interventions that address identified gaps. Additionally, for equitable implementation of telehealth, systems or clinics must identify the characteristics of their catchment area that go beyond individual factors (eg, space to complete movement assessments in the home, privacy for web-based connections with physical therapists) to include care delivery constraints (eg, beginning sessions late and running over time of scheduled appointment). Patient, clinical providers, health care systems, and communities need a shared mental model of such adaptations to collectively understand the role and impact of changes on telehealth access, receipt, and delivery. Holtrop et al [35] provide a useful table describing methods to elicit mental models. Understanding mental models can help to select potential implementation strategies needed to promote the equitable implementation of telehealth across a variety of disciplines [35,58,59].

Finally, as we approach postpandemic implementation of telehealth, building a *digital infrastructure* has the potential to mitigate long-standing issues with the inverse relationship between the need for health care and use or access [55] across different populations. Building a digital infrastructure requires attention to engagement, access, training (including cultural humility), and equity-focused measurement [25,60,61]. Future research needs to evaluate the cost-effectiveness of telehealth that includes policy analysis and program evaluation related to the construction and sustainment of digital infrastructure in communities.

## Conclusions

Enhancing equitable implementation of telehealth is timely and critical to advancing the health and well-being of *all* persons. The tension between ongoing innovation in telehealth that is occurring in the context of the evolving pandemic creates opportunities for innovation *and* unanticipated challenges to equitable implementation. Equity frameworks help connect internal and external contexts that create disparities and to consider the implementation strategies that may address them. Bridging factors such as community partnerships, shared mental models, and digital infrastructure can guide implementation, adaptations, and sustainability in the setting of a rapidly changing landscape for telehealth.

## Abbreviations

DHEF: Digital Health Equity Framework
EPIS: Exploration, Preparation, Implementation, and Sustainment
